# Condensed Mitochondria Assemble Into the Acrosomal Matrix During Spermiogenesis

**DOI:** 10.3389/fcell.2022.867175

**Published:** 2022-04-21

**Authors:** Mindong Ren, Yang Xu, Colin K. L. Phoon, Hediye Erdjument-Bromage, Thomas A. Neubert, Sujith Rajan, M. Mahmood Hussain, Michael Schlame

**Affiliations:** ^1^ Departments of Anesthesiology, New York, NY, United States; ^2^ Department of Cell Biology, New York, NY, United States; ^3^ Department of Pediatrics, New York, NY, United States; ^4^ Kimmel Center for Biology and Medicine at the Skirball Institute, New York University School of Medicine, New York, NY, United States; ^5^ Department of Foundations of Medicine, NYU Long Island School of Medicine, Mineola, NY, United States

**Keywords:** acrosome, cristae, mitochondria, spermatogenesis, spermiogenensis

## Abstract

Mammalian spermatogenesis is associated with the transient appearance of condensed mitochondria, a singularity of germ cells with unknown function. Using proteomic analysis, respirometry, and electron microscopy with tomography, we studied the development of condensed mitochondria. Condensed mitochondria arose from orthodox mitochondria during meiosis by progressive contraction of the matrix space, which was accompanied by an initial expansion and a subsequent reduction of the surface area of the inner membrane. Compared to orthodox mitochondria, condensed mitochondria respired more actively, had a higher concentration of respiratory enzymes and supercomplexes, and contained more proteins involved in protein import and expression. After the completion of meiosis, the abundance of condensed mitochondria declined, which coincided with the onset of the biogenesis of acrosomes. Immuno-electron microscopy and the analysis of sub-cellular fractions suggested that condensed mitochondria or their fragments were translocated into the lumen of the acrosome. Thus, it seems condensed mitochondria are formed from orthodox mitochondria by extensive transformations in order to support the formation of the acrosomal matrix.

## Introduction

Spermatogenesis triggers a series of striking changes in the intracellular composition and the spatial arrangement of organelles (Jan et al., 2012; Kotaja, 2013). Among those changes are profound alterations in the morphology of mitochondria and a shift from glycolytic to oxidative energy metabolism. The final products of the mitochondrial transformation are sperm mitochondria that have ultra-high respiratory capacity (Meinhardt et al., 1999; Rato et al., 2012; Amaral et al., 2013; Ramalho-Santos and Amaral, 2013; Varuzhanyan and Chan, 2020). While the shift from low to high respiration fits the general paradigm that cellular differentiation stimulates the development of cristae and the expression of oxidative enzymes (Lisowski et al., 2018), the changes observed in male germ cells are far more complex. For instance the fission-fusion equilibrium of mitochondria, which has been shown to be critical for spermatogenesis (Varuzhanyan et al., 2019), progresses towards fusion during meiosis, then towards fission in the early post-meiotic phase, and finally towards fusion again in the late post-meiotic phase (Varuzhanyan and Chan, 2020). Even more puzzling are the changes in mitochondrial ultrastructure. Since the pioneering studies of the late 1970s, it has been recognized that meiosis leads to an expansion of the intermembrane space at the expense of the matrix (De Martino et al., 1979). The most advanced stage of this transformation is a unique type of mitochondria, in which the matrix is reduced to a narrow shell surrounding a large central cavity. This form was called “condensed” because it bears some resemblance to the condensed state of isolated liver mitochondria during active respiration (Hackenbrock, 1966; Hackenbrock, 1968).

However, the true nature of condensed mitochondria has remained obscure. It is not known whether they represent a reversible functional state or a distinct mitochondrial population. Condensed germ cell mitochondria were found to be resistant to changes in osmotic pressure (De Martino et al., 1979), which makes them different from condensed liver mitochondria (Hackenbrock, 1966; Hackenbrock, 1968). This challenges the idea that condensed mitochondria merely represent the actively respiring state of a single population of germ cell mitochondria. Although condensed mitochondria were shown to respire (De Martino et al., 1979), they were not compared side-by side to orthodox mitochondria, which leaves the magnitude of their respiratory capacity unknown. Finally, it is not clear why condensed mitochondria appear only transiently, how they are removed from the cell, and what their function is.

We recently discovered that in addition to energy metabolism, germ cell mitochondria contribute to the biogenesis of acrosomes, a large vesicle with a cap-like shape that sits on top of the nucleus near the apex of sperm (Ren et al., 2019). Acrosomes are formed during the Golgi and Cap phases of spermiogenesis and are critical to fertilization (Berruti, 2016; Khawar et al., 2019). Here we provide evidence that condensed mitochondria are distinct organelles with a stable ultrastructure and a distinguishable proteome and we show that they are assembled into the lumen of the acrosome.

## Materials and Methods

### Mice

All protocols were approved by the Institutional Animal Care and Use Committee of the NYU School of Medicine and conform to the Guide for the Care and Use of Laboratory Animals published by the National Institutes of Health (NIH). Mice were housed under temperature-controlled conditions using a 12-h light/dark cycle with free access to drinking water and food. The *Ant4-KO* strain was developed by the laboratory of N. Terada (Brower et al., 2007). The mouse line was re-derived in our laboratory by *in vitro* fertilization of C57BL/6 eggs with sperm from heterozygous *Ant4+/−* males. The heterozygous animals were bred to produce homozygous *Ant4-KO* mice. Genotyping was performed by PCR analysis of genomic DNA as previously described (Brower et al., 2007).

### Transmission Electron Microscopy

Testes of mice were de-capsulated and fixed with 2.5% glutaraldehyde and 2% paraformaldehyde in 0.1M sodium cacodylate buffer (pH 7.2) for 2 h. After treatment with 1% osmium tetroxide for 1 h, the testes were stained with 1% uranyl acetate aqueous solution overnight at 4°C. The samples were rinsed in water, dehydrated in graded series of ethanol, infiltrated with propylene oxide/EMbed 812 mixtures and embedded in EMbed 812 resin (Electron Microscopy Sciences, PA, United States). Ultrathin sections (70 nm for morphology or 200 nm for tomography) were cut, mounted on formvar coated slot grids and stained with uranyl acetate and lead citrate by standard methods. Gold fiducials (15 nm) were added and a thin layer of carbon was deposited to the sample side of the grid for tomography. Imaging was performed by a Talos120C transmission electron microscope (Thermo Fisher Scientific, Hillsboro, OR) and recorded using Gatan (4 k × 4 k) OneView Camera with software Digital Micrograph (Gatan Inc., Pleasanton, CA). Random images were collected at different magnifications. First, the developmental stage of seminiferous tubules was determined at low magnification. Then, individual germ cells were identified based on their morphology and their proximity to the lumen and the basal membrane, respectively (Russell et al., 1990). Finally, quantitative analyses of germ cell mitochondria were performed at a magnification of 4,000–9,000. Morphometric features of mitochondria were measured at a magnification of 10,000–15,000. Images were analyzed with the software ImageJ.

### Collection and Analysis of Tomograms

Samples were tilted from −70 and +70° at two-degree intervals. The tilt series were collected with a high-tilt tomography holder (Gatan Model 916) using Serial EM program for automated data collection (Mastronarde 2005). A second tilt series of the same area was collected after manually rotating the specimen support by ninety degrees. Dual-axis tomograms were reconstructed by IMOD (Kremer et al., 1996). Segmentations were generated by ORS Dragonfly 4.1 (ORS Object Research Systems, Montreal, QC).

### Immunogold Labeling

Testis sections (100 nm thickness) were placed on carbon formvar 200 mesh and incubated for 5 min in phosphate-buffered saline (PBS) containing 1% bovine serum albumin, 0.05% Triton X-100, and 0.05% Tween 20. The primary antibody (rabbit polyclonal antibody to ANT4, #PA5-44133 from Thermo-Fisher) was added in several dilutions and incubated with the sections overnight at 4°C. Sections were washed six times with PBS and incubated with the secondary antibody (18 nm gold-conjugated goat anti-rabbit secondary antibody) at a dilution of 1:15 in PBS containing 1% bovine serum albumin, 0.05% Triton X-100, and 0.05% Tween 20 for 30–60 min. After washing six times with PBS, sections were fixed with 2% paraformaldehyde in PBS for 5 min. The sections were washed four times with water and stained with 3% uranyl acetate for 5 min in the dark. Sections were washed six times with water and then stained with lead acetate for 1 min. Finally, the sections were washed again six times.

### Isolation of Mitochondria and Cytoplasmic Vesicles

Mitochondria were isolated from mouse testis and liver in ice-cold isolation medium containing 0.28 M sucrose, 10 mM Tris, pH 7.4, and 0.25 mM EDTA. Four de-capsulated testes were homogenized in 12 ml isolation medium using a tight-fitting Teflon-glass homogenizer. The homogenate was spun at 700 g for 10 min. After that, the supernatant was spun at 8,000 g for 15 min. The pellet was resuspended in isolation medium at a protein concentration of ∼10 g/L. Protein concentrations were determined by the Lowry procedure (Lowry et al., 1951). The 8,000-g pellet was loaded onto a 30% Percoll solution in isolation medium and spun at 95,000 g for 30 min in a swing-out rotor. This step separated the mitochondria into a low-density and a high density band. Those bands were collected and diluted 1:8 with isolation medium. The low- and high-density mitochondria were pelleted by centrifugation at 10,000 g for 20 min. Condensed mitochondria were immuno-isolated from the low-density fraction and orthodox mitochondria were immuno-isolated from the high-density fraction, using the Mitochondria Isolation Kit (Miltenyi Biotec; 130-096-946). Briefly, samples were incubated with anti-Tom22–coated microbeads. The monoclonal anti-Tom22 antibody specifically binds Tom22 of mouse mitochondria. Next, each sample was loaded onto a MACS column placed in the magnetic field of the MACS Separator, by which the microbeads and the bound mitochondria were retained on the column, whereas the unbound organelles were allowed to pass through. Cytoplasmic vesicles were collected by centrifugation from the flow-through of the low-density fraction. The magnetically retained mitochondria were eluted from the column after it was removed from the magnetic field. Mouse liver mitochondria were isolated from liver homogenate by differential centrifugation as described above without density gradient or affinity purification.

### Sub-Fractionation of Acrosomes

Sperm isolated from murine cauda epididymis were capacitated in HEPES (10 mM, pH7.4)-buffered DMEM supplemented with bovine serum albumin (BSA, 20 g/L) and CaCl_2_ (2 mM) for 45 min at 37°C. After sedimentation (500 g, 10 min) and resuspension in BSA-free HEPES-buffered DMEM with CaCl_2_ (2 mM), the capacitated sperm were treated with the calcium ionophore A23187 (10 µM) for 30 min at 37°C to initiate the acrosome reaction. After centrifugation for 5 min at 1,000 g to pellet the post-reaction residual sperm, the supernatant containing the released acrosomal vesicles and matrix was centrifuged at 100,000 g for 1 h at 4°C. The pellet was resuspended in a small volume of ice-cold PBS using a Dounce homogenizer and laid over a step gradient consisting of 0.3, 0.5, 0.7, 0.9, 1.1, 1.3, and 1.5 M sucrose in PBS. After overnight centrifugation (200,000 g at 4°C), seven equal-volume fractions were collected from the top to the bottom of the sucrose gradient. These fractions were processed for Western blotting and lipidomic and proteomic analyses.

### Protease Treatment of Mitochondria

To determine the submitochondrial localization of ANT4, the Percoll density gradient-purified heavy (orthodox) and light (condensed) mitochondria from mouse testis were resuspended to a protein concentration of 750 μg/ml in ice-cold isolation medium containing 0.28 M sucrose, 10 mM Tris pH 7.4, and 0.25 mM EDTA. Samples were treated with pronase E (250 μg/ml), digitonin (2.5 mg/ml), or Triton X-100 (0.5%) for 30 min on ice, after which protease digestion was halted by the addition of PMSF (5 mM). Proteins were lysed in SDS sample buffer and analyzed by SDS-PAGE and immunoblotting.

### Measurement of Mitochondrial Respiration

The oxygen consumption rate of isolated mitochondria was measured in an Agilent Seahorse XFe24 Analyzer according to the manufacturer’s instructions (Application Note 5991-7145EN, 2016) with slight modifications. Briefly, the isolation medium (0.28 M sucrose, 10 mM Tris pH 7.4, and 0.25 mM EDTA) containing 0.5% (w/v) fatty acid-free BSA was used throughout the mitochondrial isolation procedure. Crude testis mitochondria (8,000 g pellet) were further fractionated on a 30% Percoll gradient into heavy and light mitochondria for the Seahorse assay. After removal of Percoll, heavy and light mitochondria from testis as well as mitochondria from liver were resuspended in a minimal volume of ice-cold isolation medium without BSA. Total protein concentration was determined using the Bradford Assay reagent (Bio-Rad). For the Seahorse assay, mitochondria were first diluted to a protein concentration of 0.8 g/L in ice-cold mitochondrial assay solution containing 70 mM sucrose, 220 mM mannitol, 10 mM KH_2_PO_4_, 5 mM MgCl_2_, 2 mM HEPES, 1.0 mM EGTA, 0.2% fatty acid-free BSA, pH 7.2, 5 mM succinate, 5 mM malate, 5 mM glutamate, and 5 mM pyruvate. Aliquots of 50 μL of mitochondrial suspension (40 μg protein) were applied to each well of the XF24 cell culture plate. The plate was then transferred to a centrifuge equipped with a swinging bucket microplate adaptor and spun at 2,000 g for 20 min at 4°C. After centrifugation, 450 μL of pre-warmed (37°C) mitochondrial assay solution was added to each well and the plate was transferred to the Seahorse XFe24 Analyzer to initiate the measurement. Oxygen consumption rates were determined before and after sequential addition of ADP (4 mM), oligomycin (2.5 μg/ml), FCCP (4 μM), and rotenone (2 μM) plus antimycin A (4 μM).

### Cardiolipin Analysis

Cardiolipin was analyzed in subcellular fractions by LC-ESI-MS/MS on a QExactive HF-X instrument coupled directly to a Vanquish UHPLC (Thermo Scientific). Lipids were extracted (Bligh and Dyer, 1959) and a 7 µL aliquot was injected into a Restek Ultra C18 reversed-phase column (100 × 2.1 mm; particle size 3 µm) that was kept at a temperature of 50°C. Chromatography was performed with solvents A and B at a flow rate of 0.15 ml/min. Solvent A contained 600 ml acetonitrile, 399 ml water, 1 ml formic acid, and 0.631 g ammonium formate. Solvent B contained 900 ml 2-propanol, 99 ml acetonitrile, 1 ml formic acid, and 0.631 g ammonium formate. The chromatographic run time was 40 min, changing the proportion of solvent B in a non-linear gradient from 30 to 35% (0–2 min), from 35 to 67% (2–5 min), from 67 to 83% (5–8 min), from 83 to 91% (8–11 min), from 91 to 95% (11–14 min), from 95 to 97% (14–17 min), from 97 to 98% (17–20 min), from 98 to 100% (20–25 min), and from 100 to 30% (25–26 min). For the remainder of the run time the proportion of solvent B stayed at 30% (26–40 min). The mass spectrometer was operated in negative ion mode. The spray voltage was set to 4 kV and the capillary temperature was set to 350°C. MS1 scans were acquired at a resolution of 120,000, an AGC target of 1e6, a maximal injection time of 65 ms, and a scan range of 300–2000 m/z. MS2 scans were acquired at a resolution of 30,000, an AGC target of 3e6, a maximal injection time of 75 ms, a loop count of 11, and an isolation window of 1.7 m/z. The normalized collision energy was set to 30 and the dynamic exclusion time to 13 s. For lipid identification and quantitation, data were analyzed by the software LipidSearch 4.1 SP1 (Thermo Scientific). The general database of LipidSearch was searched with a precursor tolerance of 2 ppm, a product tolerance of 0.2 Da, an intensity threshold of 1.0%, and an m-score threshold of 5.

### 2D-BN-PAGE and Western Blotting

Mitochondria (0.2 mg of protein) were solubilized with digitonin (8 g/g protein) and separated by Blue Native/SDS–PAGE (Wittig et al., 2006). The solubilization buffer contained 50 mM NaCl, 50 mM imidazole, 2 mM aminohexanoic acid, and 1 mM EDTA (pH 7.0). Samples were spun at 100,000 g for 15 min, and solubilized proteins were collected from the supernatant. Coomassie Blue G-250 was added, and the proteins were separated by electrophoresis on a 4–13% gradient Blue Native gel. Gel strips were cut, soaked in 1% SDS for 15 min at 37°C, and horizontally placed on top of a 10% SDS–PAGE gel (10% T, 3% C). After the second electrophoresis, proteins were transferred to a PVDF membrane for Western blotting. PVDF membranes were incubated with primary monoclonal antibodies (1 μg/ml) in Odyssey blocking buffer containing 0.01% Tween-20. Primary antibodies included mouse monoclonal antibodies to the α-subunit of ATP synthase and to subunit I of cytochrome oxidase (Abcam). Fluorescent LiCor GAM-IRDye680 secondary antibodies were used at a dilution of 1:15,000. Proteins were visualized and quantified by the LiCor scanner.

### Proteomics

Sample proteins were concentrated by short SDS-PAGE runs into single 1 cm bands that contained all proteins before tryptic digestion and peptide extraction. Tandem mass tag (TMT)-labeling and the remaining proteomics protocols were performed by using methods previously described (Huang et al., 2017; Erdjument-Bromage et al., 2018) with the following modifications. TMT Label 129N, 129C, 130N, and 130C were added to each sample at a label:peptide ratio of 12:1 (wt/wt) and mixed briefly on a vortexer. The mixture was incubated at room temperature for 1 h, quenched by the addition of 10 μL 5% hydroxylamine, and then acidified by the addition of 10 μL 10% formic acid. A small aliquot from each reaction was desalted with Empore C18 High Performance Extraction Disks, and the eluted peptide solutions were partially dried under vacuum and then analyzed by LC-MS/MS with a Q Exactive High Field Orbitrap mass spectrometer to determine labeling efficiencies, which were found to be 98–99%. To determine protein amounts, samples were mixed and analyzed in test runs by LC-MS/MS. The final sample mixture containing mixed channels was prepared by adjusting the volume of individual samples so they contained equal amounts of labeled peptides. The mixture was desalted by using a Sep-Pak tC18 1 cc Vac Cartridge (Waters; WAT036820). Eluted peptides were subjected to triplicate analysis by using LC with a Thermo Easy nLC 1,000 system coupled online to a Q Exactive HF with a NanoFlex source (Thermo Fisher Scientific) as previously described (Huang et al., 2017). All data were analyzed by MaxQuant proteomics software (version 1.5.5.1) with the Andromeda search engine (Cox et al., 2011) using a mouse database [mouse (*Mus musculus*) protein database; Uniprot; Reviewed, 16,950 entries, (12202017)]. Reporter ion mass tolerance was set to 0.01 D, the activated precursor intensity fraction value was set to 0.75, and the false discovery rate was set to 1% for protein, peptide-spectrum match, and site decoy fraction levels. Peptides were required to have a minimum length of seven amino acids and a mass no greater than 4,600 D. The reporter ion intensities were defined as intensities multiplied by injection time (to obtain the total signal) for each isobaric labeling channel summed over all MS/MS spectra as previously validated (Tyanova et al., 2016). We also performed separate analyses of unlabeled peptides by using the same LC-MS/MS method. Mass spectra were subjected to label-free quantitation by using MaxQuant proteomics data analysis workflow (version 1.5.5.1) with the Andromeda search engine (Cox et al., 2011; Tyanova et al., 2016).

## Results

### Male Germ Cells Contain Structurally and Functionally Distinct Types of Mitochondria

**FIGURE 1:**
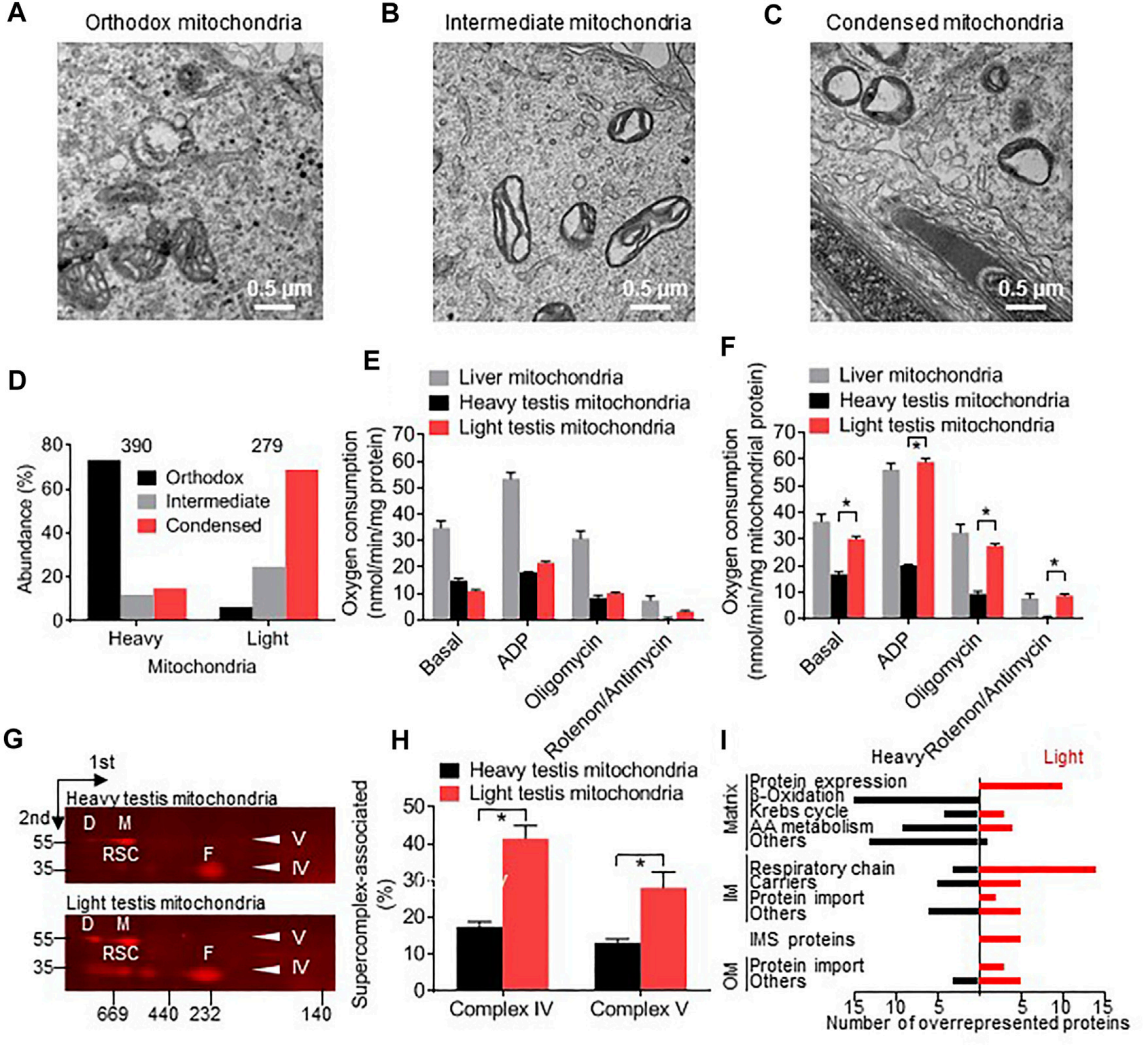
Testicular germ cells contain distinct types of mitochondria. **(A–C)**: Electron micrographs of mouse testis sections show orthodox, intermediate, and condensed mitochondria. **(D)**: Mitochondria were isolated from mouse testis, separated into heavy and light fractions by Percoll density gradient centrifugation, and affinity-purified with Tom22 antibodies. The proportion of orthodox, intermediate, and condensed mitochondria was determined by electron microscopy. The number of analyzed mitochondria is given on top. Heavy and light fractions contain different proportions of orthodox, intermediate, and condensed mitochondria (*p* < 0.0001, Chi-square test). **(E,F)**: Respiratory activities of heavy and light testis mitochondria and liver mitochondria were measured in the presence and absence of ADP and different inhibitors. Oxygen consumption was calculated per total protein **(E)** or per mitochondrial protein **(F)**. The contribution of mitochondrial protein to total protein was determined by quantitative mass spectrometry. Data are means ± SEM (*N* = 6). Asterisks indicate a significant difference between heavy and light testis mitochondria (*p* < 0.000001, t-test). **(G,H)**: The abundance of supercomplexes was measured in heavy and light testis mitochondria by 2D-BN-PAGE. Blots were probed with antibodies to MTCOI of complex IV revealing free complex IV **(F)** and respiratory supercomplexes (RSC). Blots were probed with antibodies to ATP5F1A of complex V revealing the monomer (M) and the dimer (D) of complex V. Molecular weight markers of 35 and 55 kDa are shown. Bar graphs show means ± SEM (*N* = 3). Asterisks indicate a significant difference between heavy and light testis mitochondria (*p* < 0.05, t-test). **(I)**: The protein composition of heavy and light mitochondria was compared by tandem-mass-tag proteomics. The black columns show the number of proteins that are relatively overrepresented in heavy mitochondria (heavy/light tag ratio >1 standard deviation above the mean). The red columns show the number of proteins that are relatively overrepresented in light mitochondria (heavy/light tag ratio >1 standard deviation below the mean). IM, inner membrane; OM, outer membrane.

We identified three morphologic phenotypes among germ cell mitochondria in mouse testis. These included mitochondria with small lamellar or vesicular cristae (Figure 1A), mitochondria with enlarged cristae (Figure 1B), and mitochondria with a single vacuole-like crista (Figure 1C). In keeping with the terminology introduced by De Martino et al. (De Martino et al., 1979), we refer to them as “orthodox mitochondria” if multiple small cristae are surrounded by a compact matrix space and we refer to them as “condensed mitochondria” if a single crista is surrounded by a narrow matrix shell. For organelles in between the orthodox and the condensed state, we choose the term “intermediate mitochondria”.

In order to determine whether orthodox and condensed mitochondria have distinct protein compositions and are functionally different from each other, we collected mitochondria from testis homogenates, separated them by Percoll density gradient centrifugation, and affinity-purified them. As expected from their appearance in electron micrographs, condensed mitochondria accumulated in the low-density fraction whereas orthodox mitochondria accumulated in the high-density fraction (Figure 1D). Both light (condensed) and heavy (orthodox) mitochondria exhibited canonical mitochondrial activities, such as ADP-stimulated respiration that could be inhibited by oligomycin and by rotenone + antimycin. The respiratory activities of orthodox and condensed testis mitochondria were about equal to each other but were substantially lower than the respiratory activity of liver mitochondria (Figure 1E). However, condensed testis mitochondria contained more contaminating non-mitochondrial proteins than orthodox testis mitochondria, as determined by proteome analysis. Thus, the respiratory activity of condensed testis mitochondria, expressed per mitochondrial protein, was higher than the respiratory activity of orthodox testis mitochondria and similar to the respiratory activity of liver mitochondria (Figure 1F).

Next we analyzed proteins in orthodox and condensed mitochondria. Condensed mitochondria contained a higher proportion of complex IV in respiratory supercomplexes and a higher proportion of complex V in the dimer state than orthodox mitochondria (Figures 1G,H). Comparison of the protein compositions by tandem-mass-tag (TMT) proteomics revealed that 58 out of 379 mitochondrial proteins were more abundant in condensed mitochondria and 60 proteins were more abundant in orthodox mitochondria (Table 1). Proteins more abundant in condensed mitochondria belonged mostly to the respiratory chain, the mitochondrial protein expression system, the outer membrane, and the intermembrane space. In contrast, proteins more abundant in orthodox mitochondria belonged mostly to the mitochondrial matrix (Figure 1I). The only matrix proteins more abundant in condensed than in orthodox mitochondria were ribosomal proteins and t-RNA ligases (Figure 1I; Table 1), suggesting that condensed mitochondria actively assemble complexes of oxidative phosphorylation.

**TABLE 1:** List of proteins with a statistically significant difference in abundance between heavy (orthodox) and light (condensed) mitochondria. Mitochondria were isolated from mouse testis, separated into light and heavy fractions by Percoll density gradient centrifugation, and affinity-purified with TOM22 antibodies. Peptides of the two samples were differentially labeled, mixed 1:1, and analyzed by tandem-mass-tag proteomics.

Proteins more abundant in heavy mitochondria than in light mitochondria (z of log2light/heavy < −1)				Proteins more abundant in light mitochondria than in heavy mitochondria (z of log2light/heavy > +1)				
Compartment	Protein	Gene product	Function	Compartment	Protein	Gene product	Function	—
Matrix	Acyl-CoA synthetase family member 2, mitochondrial	Acsf2	FA degradation	Matrix	39S ribosomal protein L3, mitochondrial	Mrpl3	Prot synth	—
	Hydroxyacyl-coenzyme A dehydrogenase, mitochondrial	Hadh	FA degradation		39S ribosomal protein L20, mitochondrial	Mrpl20	Prot synth	—
	Medium-chain specific acyl-CoA dehydrogenase, mitochondrial	Acadm	FA degradation		Aspartate--tRNA ligase, mitochondrial	Dars2	Prot synth	—
	Short-chain specific acyl-CoA dehydrogenase, mitochondrial	Acads	FA degradation		39S ribosomal protein L49, mitochondrial	Mrpl49	Prot synth	—
	Isovaleryl-CoA dehydrogenase, mitochondrial	Ivd	FA degradation		39S ribosomal protein L22, mitochondrial	Mrpl22	Prot synth	—
	Long-chain specific acyl-CoA dehydrogenase, mitochondrial	Acadl	FA degradation		Alanine--tRNA ligase, mitochondrial	Aars2	Prot synth	—
	Acyl-CoA synthetase short-chain family member 3, mitochondrial	Acss3	FA degradation		39S ribosomal protein L40, mitochondrial	Mrpl40	Prot synth	—
	Short/branched chain specific acyl-CoA dehydrogenase, mitochondrial	Acadsb	FA degradation		39S ribosomal protein L37, mitochondrial	Mrpl37	Prot synth	—
	Lipoamide acyltransferase component of branched-chain alpha-keto acid dehydrogenase complex, mitochondrial	Dbt	FA degradation		39S ribosomal protein L16, mitochondrial	Mrpl16	Prot synth	—
	Propionyl-CoA carboxylase beta chain, mitochondrial	Pccb	FA degradation		Probable arginine--tRNA ligase, mitochondrial	Rars2	Prot synth	—
	Enoyl-CoA hydratase, mitochondrial	Echs1	FA degradation		L-2-hydroxyglutarate dehydrogenase, mitochondrial	L2hgdh	AA metabol	—
	2,4-dienoyl-CoA reductase, mitochondrial	Decr1	FA degradation		Glutaminase kidney isoform, mitochondrial	Gls	AA metabol	—
	Acetyl-CoA acetyltransferase, mitochondrial	Acat1	FA degradation		Hydroxyacylglutathione hydrolase, mitochondrial	Hagh	AA metabol	—
	Acetyl-coenzyme A synthetase 2-like, mitochondrial	Acss1	FA degradation		Glycine dehydrogenase (decarboxylating), mitochondrial	Gldc	AA metabol	—
	3-ketoacyl-CoA thiolase, mitochondrial	Acaa2	FA degradation		Pyruvate dehydrogenase E1 component subunit alpha, testis-specific form, mitochondrial	Pdha2	Krebs	—
	Succinyl-CoA ligase [GDP-forming] subunit beta, mitochondrial	Suclg2	Krebs		Probable isocitrate dehydrogenase (NAD) gamma 2, mitochondrial	—	Krebs	—
	Fumarate hydratase, mitochondrial	Fh	Krebs		ATP-citrate synthase	Acly	Krebs	—
	Isocitrate dehydrogenase [NAD] subunit gamma 1, mitochondrial	Idh3g	Krebs		Malonyl-CoA-acyl carrier protein transacylase, mitochondrial	Mcat	FA synthesis	—
	Isocitrate dehydrogenase [NADP], mitochondrial	Idh2	Krebs		—	—	—	—
	Hydroxymethylglutaryl-CoA synthase, mitochondrial	Hmgcs2	AA metabol	Inner membrane	Mimitin, mitochondrial	Ndufaf2	OXPHOS	Complex I
	Branched-chain-amino-acid aminotransferase, mitochondrial; Branched-chain-amino-acid aminotransferase	Bcat2	AA metabol		Cytochrome b-c1 complex subunit 9	Uqcr10	OXPHOS	Complex III
	4-hydroxy-2-oxoglutarate aldolase, mitochondrial	Hoga1	AA metabol		Cytochrome b-c1 complex subunit 10	Uqcr11	OXPHOS	Complex III
	4-aminobutyrate aminotransferase, mitochondrial	Abat	AA metabol		Cytochrome b	mt-Cytb; Mt-Cyb	OXPHOS	Complex III
	Methylmalonyl-CoA mutase, mitochondrial	Mut	AA metabol		Cytochrome b-c1 complex subunit 6, mitochondrial	Uqcrh	OXPHOS	Complex III
	Isobutyryl-CoA dehydrogenase, mitochondrial	Acad8	AA metabol		Cytochrome c oxidase subunit 2	mt-Co2; Mtco2	OXPHOS	Complex IV
	Glutamate dehydrogenase 1, mitochondrial	Glud1	AA metabol		Cytochrome c oxidase subunit 6B2	Cox6b2	OXPHOS	Complex IV
	Methylcrotonoyl-CoA carboxylase beta chain, mitochondrial	Mccc2	AA metabol		Cytochrome c oxidase subunit 7A2, mitochondrial	Cox7a2	OXPHOS	Complex IV
	Methylcrotonoyl-CoA carboxylase subunit alpha, mitochondrial	Mccc1	AA metabol		Cytochrome c oxidase subunit 1	mt-Co1; Mtco1	OXPHOS	Complex IV
	Aldehyde dehydrogenase, mitochondrial	Aldh2	Ethanol metabol		Cytochrome c oxidase subunit 6A, mitochondrial; Cytochrome c oxidase subunit 6A1, mitochondrial	Cox6a1	OXPHOS	Complex IV
	Aldehyde dehydrogenase X, mitochondrial	Aldh1b1	Ethanol metabol		Cytochrome c oxidase subunit 5B, mitochondrial	Cox5b	OXPHOS	Complex IV
	GTP:AMP phosphotransferase AK3, mitochondrial	Ak3	Nucleotide metabol		Cytochrome c oxidase subunit 5A, mitochondrial	Cox5a	OXPHOS	Complex IV
	Methylmalonate-semialdehyde dehydrogenase (acylating), mitochondrial	Aldh6a1	Pyrimidine metabolism		—	Cox7b2	OXPHOS	Complex IV
	Succinate-semialdehyde dehydrogenase, mitochondrial	Aldh5a1	GABA metabol		HIG1 domain family member 1A, mitochondrial	Higd1a	OXPHOS	Complex IV
	Methylglutaconyl-CoA hydratase, mitochondrial	Auh	Metabol		Mitochondrial carrier homolog 2	Mtch2	Carrier	—
	Electron transfer flavoprotein subunit beta	Etfb	Oxidoreductase		Solute carrier family 35 member F6	Slc35f6	Carrier	—
	Electron transfer flavoprotein subunit alpha, mitochondrial	Etfa	Oxidoreductase		Solute carrier family 25 member 40	Slc25a40	Carrier	—
	Adrenodoxin, mitochondrial	Fdx1	Steroid synth		Mitochondrial thiamine pyrophosphate carrier	Slc25a19	Carrier	—
	Complement component 1 Q subcomponent-binding protein, mitochondrial	C1qbp	Protein synthesis		ADP/ATP translocase 4; ADP/ATP translocase 4, N-terminally processed	Slc25a31	Carrier	—
	Peptidyl-prolyl cis-trans isomerase F, mitochondrial	Ppif	Folding		Mitochondrial import inner membrane translocase subunit TIM16	Pam16	TIM	—
	60 kDa heat shock protein, mitochondrial	Hspd1	Folding		Mitochondrial import inner membrane translocase subunit TIM50	Timm50	TIM	—
	Lon protease homolog, mitochondrial	Lonp1	Protease		Mitochondrial inner membrane protease subunit 2	Immp2l	Protease	—
	—	—	—		Phosphatidylserine decarboxylase proenzyme; Phosphatidylserine decarboxylase alpha chain; Phosphatidylserine decarboxylase beta chain	Pisd; Gm20671	PL metabol	—
Inner membrane	Cholesterol side-chain cleavage enzyme, mitochondrial	Cyp11a1	Steroid synth		Acylglycerol kinase, mitochondrial	Agk	PL metabol	—
	NADPH:adrenodoxin oxidoreductase, mitochondrial	Fdxr	Steroid synth		LETM1 domain-containing protein LETM2, mitochondrial	Letm2	Prot synth	—
	Sterol 26-hydroxylase, mitochondrial	Cyp27a1	Steroid synth		Ubiquinone biosynthesis O-methyltransferase, mitochondrial	Coq3	Ubiquinin synth	—
	ADP/ATP translocase 2; ADP/ATP translocase 2, N-terminally processed	Slc25a5	Carrier		—	—	—	—
	ADP/ATP translocase 1	Slc25a4	Carrier	Intermembrane space	Sulfite oxidase, mitochondrial	Suox	Sulfite to sulfate	—
	Solute carrier family 25 member 45	Slc25a45	Carrier		Adenylate kinase 2, mitochondrial; Adenylate kinase 2, mitochondrial, N-terminally processed	Ak2	Nucleotide metabol	—
	Calcium-binding mitochondrial carrier protein Aralar2	Slc25a13	Carrier		Serine protease HTRA2, mitochondrial	Htra2	Protease	—
	Solute carrier family 25 member 35	Slc25a35	Carrier		Cytochrome c, testis-specific	Cyct	Sperm-specific	—
	Cytochrome c oxidase subunit NDUFA4	Ndufa4	OXPHOS		Diablo homolog, mitochondrial	Diablo	Apoptosis	—
	—	Ndufv3	OXPHOS		—	—	—	—
	Cytochrome c oxidase subunit 6B1	Cox6b1	OXPHOS	Outer membrane	Mitochondrial import receptor subunit TOM22 homolog	Tomm22	TOM	—
	Electron transfer flavoprotein-ubiquinone oxidoreductase, mitochondrial	Etfdh	Ubiquinone reduction		Mitochondrial import receptor subunit TOM34	Tomm34	TOM	—
	Glycine amidinotransferase, mitochondrial	Gatm	AA metabol		Mitochondrial import receptor subunit TOM70	Tomm70a	TOM	—
	Choline dehydrogenase, mitochondrial	Chdh	Betaine synthesis		Mitochondria-eating protein	Spata18	—	—
	—	—	—		Sperm mitochondrial-associated cysteine-rich protein	Smcp	Sperm-specific	—
Outer membrane	Spermatogenesis-associated protein 19, mitochondrial	Spata19	Sperm-specific		Glycerol-3-phosphate acyltransferase 2, mitochondrial	Gpat2	PL metabol	—
	Mitochondrial amidoxime reducing component 2	Marc2	Drug metabol		Mitochondrial fission factor	Mff	Fission	—
	Voltage-dependent anion-selective channel protein 1	Vdac1	VDAC		Mitochondrial Rho GTPase 2	Rhot2	Mito distribution	—
Unassigned	Thioredoxin-dependent peroxide reductase, mitochondrial	Prdx3	Ox stress	Unassigned	Evolutionarily conserved signaling intermediate in Toll pathway, mitochondrial	Ecsit	Signaling	—
	Enoyl-CoA hydratase domain-containing protein 3, mitochondrial	Echdc3	—		—	—	—	—

In summary, we observed orthodox, intermediate, and condensed mitochondria in male germ cells and showed that they represent distinct mitochondrial populations with specific protein compositions and functional characteristics. Condensed mitochondria contain less matrix proteins and more inner and outer membrane proteins than orthodox mitochondria along with a higher amount of the import and expression system required to assemble complexes of oxidative phosphorylation. Despite their vacuole-like appearance, condensed mitochondria respire actively and contain more respiratory supercomplexes than orthodox mitochondria.

### Orthodox Mitochondria Transform Into Condensed Mitochondria During Meiosis

**FIGURE 2:**
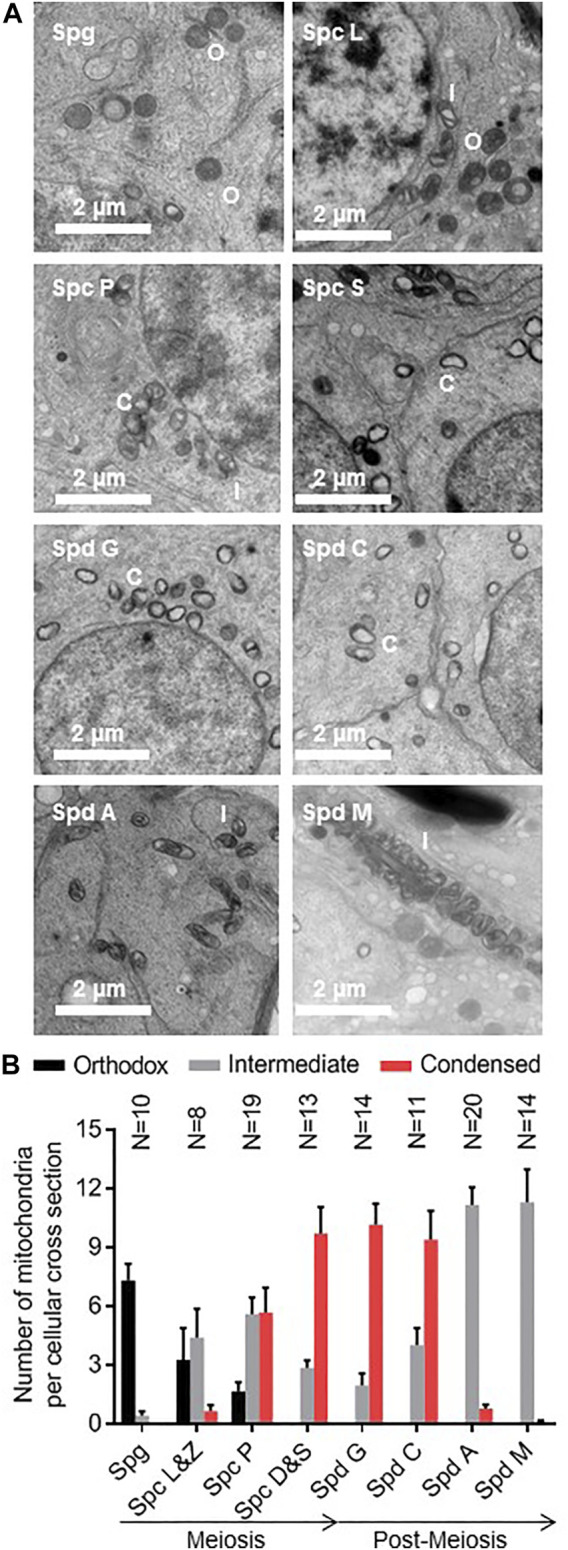
Condensed mitochondria appear transiently in meiotic and early post-meiotic germ cells. **(A)**: Testis sections of mice were analyzed by electron microscopy. The developmental stage of individual seminiferous tubules was determined and individual germ cells were identified by their distance to the basal membrane and by their morphology. Orthodox (O), intermediate (I), and condensed (C) mitochondria were identified. **(B)**: The number of orthodox, intermediate, and condensed mitochondria is shown for spermatogonia (Spg), leptotene (Spc L), zygotene (Spc Z), pachytene (Spc P), diplotene (Spc D), and secondary (Spc S) spermatocytes and spermatids in the Golgi phase (Spd G), cap phase phase (Spd C), acrosome phase (Spd A), and maturation phase (Spd M). Bar graphs represent means ± SEM. The number of analyzed cells (N) is given for each cell type.

We measured the abundance of orthodox, intermediate, and condensed mitochondria at different stages of spermatogenesis (Figure 2A). Undifferentiated germ cells (spermatogonia) contained mostly orthodox mitochondria but with the onset of meiosis, intermediate mitochondria began to appear. As meiosis progressed, condensed mitochondria appeared whereas the abundance of intermediate mitochondria decreased. Condensed mitochondria remained the most abundant species in the early post-meiotic phase. However, their number declined in the late post-meiotic phase during which they were replaced by intermediate mitochondria. The latter remained the only detectable species of mitochondria at the final stages of spermiogenesis (Figure 2B).

**FIGURE 3:**
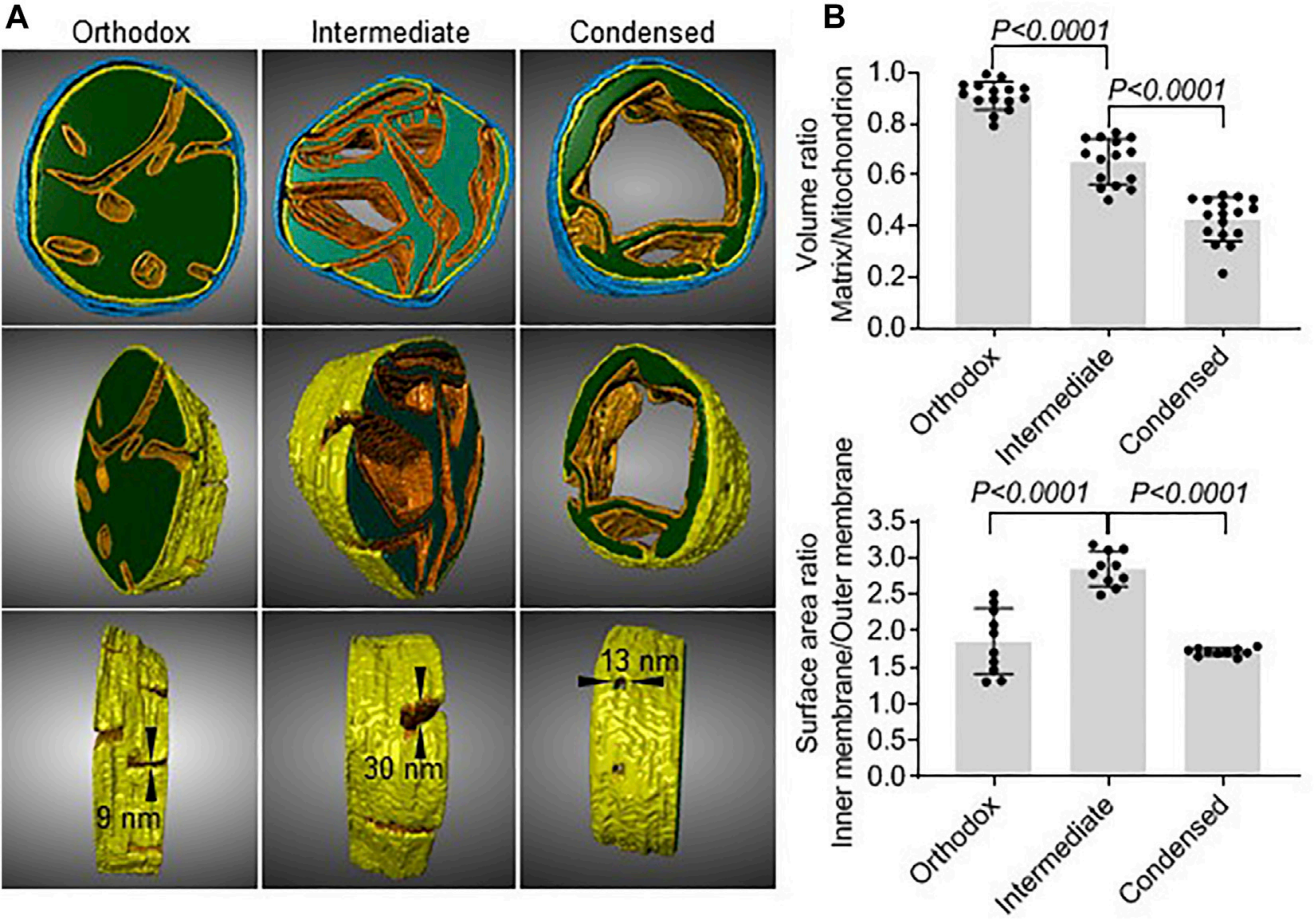
Matrix volume and inner membrane surface area change during the transformation from orthodox to condensed mitochondria. **(A)**: Electron tomograms were created of 200 nm sections of mouse testis. Image segmentation of individual mitochondria was performed manually. The matrix is shown in green, the cristae membranes are shown in orange, the inner boundary membrane is shown in yellow, and the outer membrane is shown in blue. Cristae junctions and their dimensions are indicated. **(B)**: Quantitative electron microscopy was performed in mouse testis sections. Each data point represents the measurement in an individual mitochondrion. Bar graphs show means ± SD. Means were compared by t-test.

Next, we created 3D models of the different types of mitochondria using electron tomography. They demonstrated that in orthodox mitochondria the mitochondrial matrix was a large compact body penetrated by multiple cristae. In condensed mitochondria, the matrix formed a narrow shell around a single crista. As a result, the matrix of condensed mitochondria was enclosed by two parallel sheets of inner membrane, one of which was derived from the inner boundary membrane and the other from the crista membrane. The inner membrane/matrix shell of condensed mitochondria contained holes (crista junctions) with a diameter of 13 nm (Figure 3A). Assuming that orthodox mitochondria are the precursors of intermediate mitochondria, the data suggest that the transformation requires the proliferation of cristae membranes, the enlargement of cristae junctions, and the reduction of the matrix space. Further development into condensed mitochondria requires the consolidation of cristae and an additional shrinkage of the matrix space (Figure 3A). We confirmed these results by quantitative 2D electron microscopy. The data showed that the change from orthodox to intermediate mitochondria was associated with an increase in the surface area of the inner membrane and a decrease in the matrix volume while the change from intermediate to condensed mitochondria was associated with a decrease of the surface area of the inner membrane and a further decrease in the matrix volume (Figure 3B).

In summary, our data demonstrate that orthodox mitochondria transform during meiosis first into intermediate and then into condensed mitochondria and that condensed mitochondria vanish in the post-meiotic period. They reveal the unique morphology of condensed mitochondria and suggest that condensed mitochondria evolve gradually from orthodox mitochondria by contracting the matrix volume and by first increasing and then decreasing the surface area of the inner membrane.

### Condensed Mitochondria Participate in the Assembly of Acrosomes

**FIGURE 4:**
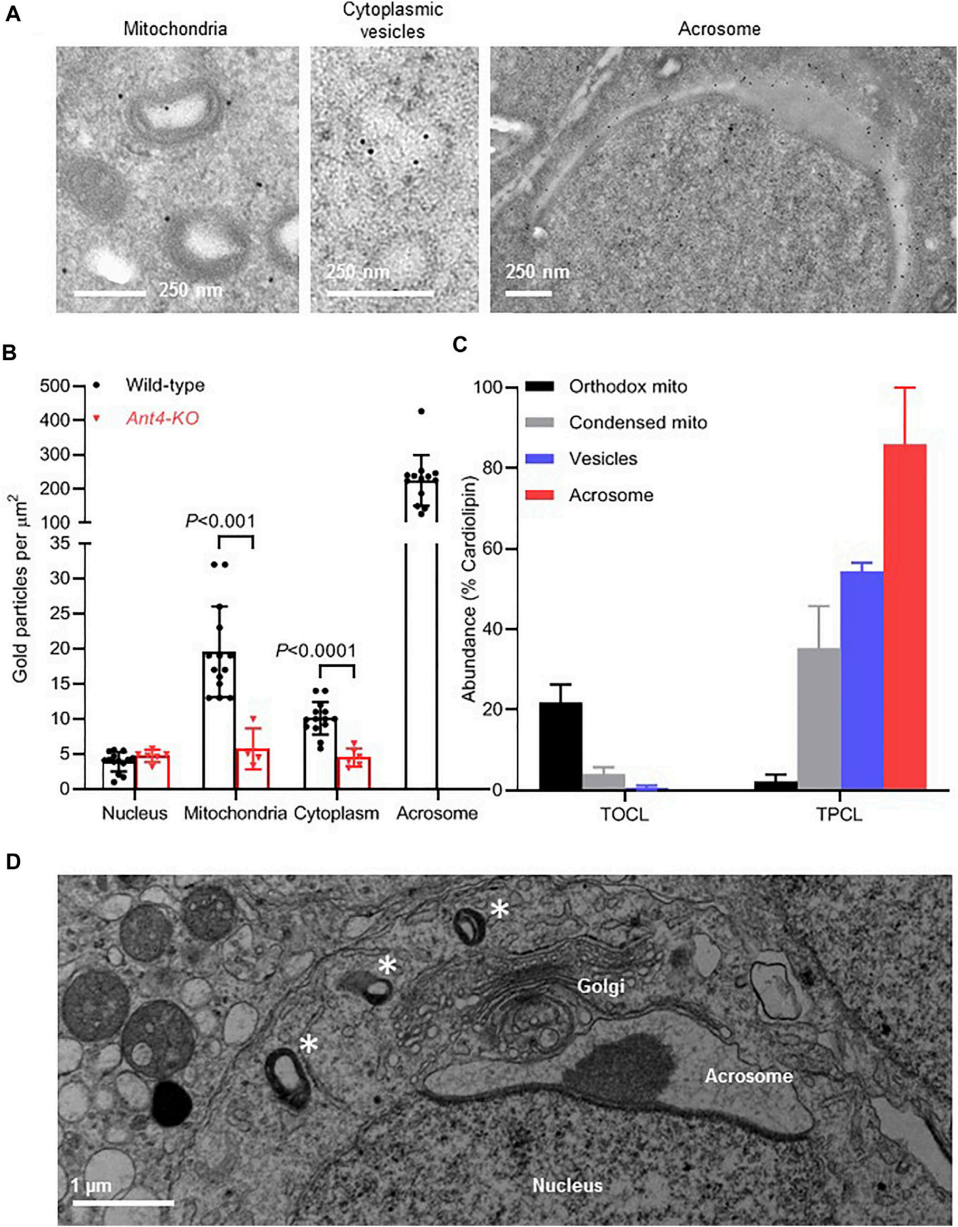
ANT4 is translocated from mitochondria to the acrosome *via* cytoplasmic vesicles. **(A)**: The localization of ANT4 was determined by immune electron microscopy in a round cap phase spermatid of wild-type mice. **(B)**: The density of Ant4 was determined in different cellular compartments by immune electron microscopy of testicular germ cells of wild-type and *Ant4-KO* mice. Each data point represents a separate electron micrograph. The bar graph shows means ± SD. Wild-type and *Ant4-KO* were compared by t-test. **(C)**: Orthodox and condensed mitochondria and cytoplasmic vesicles were purified from testis homogenate. Acrosomal material was collected from the extracellular medium of mouse sperm after the acrosome reaction. The composition of cardiolipin was analyzed by mass spectrometry in order to determine the relative abundance of TOCL and TPCL. Data are means ± SEM (*N* = 3). **(D)**: The electron micrograph shows a round spermatid with condensed mitochondria (asterisks) in the vicinity of the Golgi-acrosome-nucleus complex.

Although about 90% of mitochondria had the condensed morphology at the end of meiosis, they disappeared completely in the post-meiotic period, suggesting they were either degraded or assimilated into other cellular compartments. In order to identify compartments that harbor mitochondrial proteins, we performed immunogold labeling of ANT4, a marker protein of condensed mitochondria (Ren et al., 2019). We observed ANT4 in three different locations, including mitochondria, cytoplasmic vesicles, and the acrosome (Figure 4A). To determine whether ANT4 is an authentic resident of these compartments, we established the non-specific immunogold labeling by measuring the density of gold particles in *Ant4-knockout* (KO) mice. Low densities of about five gold particles per μm^2^ were found in all cellular compartments of *Ant4-KO* cells. In contrast, the ANT4 densities were significantly higher in mitochondria and the cytoplasm but not in the nucleus of wild-type cells. By far the highest ANT4 density was detected in the acrosome, consistent with previous data showing that acrosomes contain mitochondrial proteins (Ren et al., 2019). Background densities could not be established for the acrosome because *Ant4-KO* cells were devoid of acrosomes (Figure 4B).

The presence of ANT4 in the cytoplasm raises the possibility that condensed mitochondria release vesicles or transform into new membrane-bound organelles, which are then incorporated into the acrosome. To determine precursor-product relationships between mitochondria, cytoplasmic vesicles, and acrosomes, we analyzed cardiolipin. The cardiolipin species composition is informative in that regard because we have shown that cardiolipin is translocated from mitochondria to the acrosome and changes its species composition in the process. Whereas tetraoleoyl-cardiolipin (TOCL) is the most abundant species in mitochondria, tetrapalmitoyl-cardiolipin (TPCL) is the most abundant species in the acrosome (Ren et al., 2019). Here we found that the abundance of the mitochondria-specific TOCL decreased in the order orthodox mitochondria > condensed mitochondria > cytoplasmic vesicles > acrosome whereas the abundance of the acrosome-specific TPCL increased in the same order (Figure 4C). These data are consistent with the idea that condensed mitochondria transform into vesicles that can no longer be recognized as mitochondria. Potentially, such vesicles are the direct precursors to be incorporated into the acrosome. Regardless of the true mechanism, condensed mitochondria can often be observed in the vicinity of the Golgi-complex surrounding the nascent acrosome, which is also consistent with a role of condensed mitochondria in acrosome biogenesis (Figure 4D).

In summary, our results suggest that condensed mitochondria transform into vesicles that become precursors of the acrosome assembly.

### Acrosomes Carry Mitochondrial Molecules in the Lumen

The acrosome is a large membrane-bound organelle, the lumen of which contains both soluble and insoluble material (Buffone et al., 2008; Khawar et al., 2019). This raises the questions as to which acrosomal sub-compartment mitochondria are assembled into and whether the entire mitochondrion or only specific mitochondrial sub-compartments participate in acrosome biogenesis.

**FIGURE 5:**
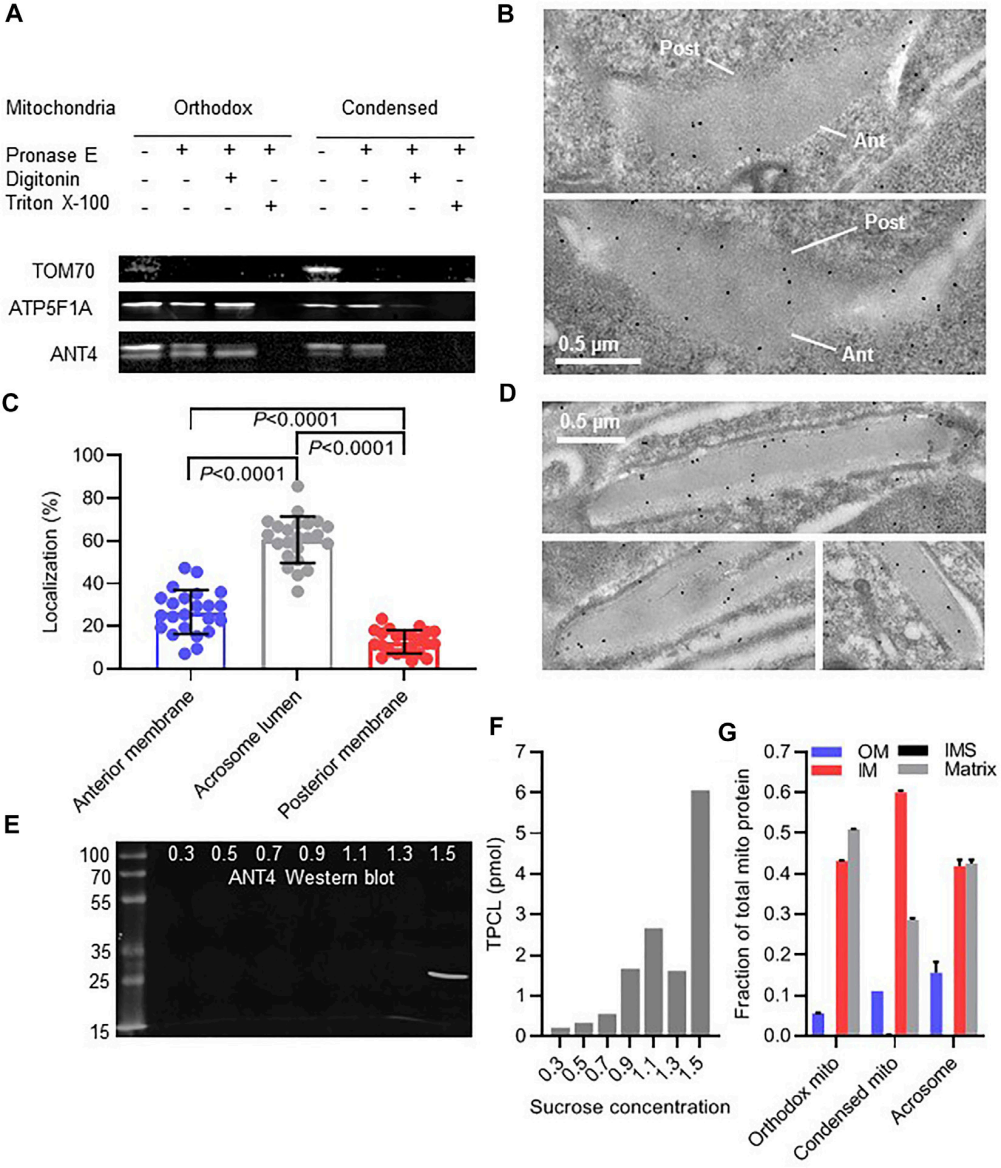
Mitochondria are incorporated into the acrosome lumen. **(A)**: Isolated mitochondria from mouse testis were treated with pronase E and digitonin or Triton X-100 followed by Western blot analysis of TOM70, ATP5F1A, and ANT4. **(B)**: The localization of ANT4 was visualized in acrosomes of round mouse spermatids by immune electron microscopy. The anterior (Ant) and posterior (Post) membranes of the acrosome are marked. **(C)**: The distribution of ANT4 within acrosomes of round spermatids was determined by quantitative immune electron microscopy. Data are means ± SD of 23 cells. Groups were compared by t-test. **(D)**: The localization of ANT4 was visualized in acrosomes of elongated mouse spermatids by immune electron microscopy. **(E,F)**: Acrosomal material was released from mouse sperm and resolved on a sucrose density gradient (0.3–1.5 M sucrose). Fractions were analyzed by Western blotting (ANT4) or by mass spectrometry (TPCL). **(G)**: Orthodox and condensed mitochondria were purified from testis homogenate. Acrosomal material was collected from the extracellular medium of mouse sperm after inducing the acrosome reaction and further purified by sucrose density gradient centrifugation. The relative abundance of mitochondrial proteins was determined by mass spectrometry. Data show the relative abundance of the outer membrane (OM), the intermembrane space (IMS), the inner membrane (IM), and the matrix, determined by summing the signal intensities of individual proteins of these compartments. Data are means ± SEM (*N* = 3).

To answer this question, we analyzed two mitochondrial markers, cardiolipin and ANT4. However, first it was necessary to establish their intramitochondrial localization. While cardiolipin is known to be associated with the inner mitochondrial membrane (Schlame et al., 2000), the localization of ANT4, a testis-specific variant of the ANT family, has not been studied. To determine the localization of ANT4 within mitochondria, we applied a classical protease protection assay to orthodox and condensed mitochondria isolated from mouse testis. As expected, the outer membrane protein TOM70 was fully degraded by pronase E in intact mitochondria whereas the matrix-facing inner membrane protein ATP5F1A was degraded only after disintegration of mitochondria by Triton X-100. ANT4 was resistant to degradation in intact mitochondria. It became susceptible to pronase E after exposure to digitonin (solubilization of the outer membrane) but required Triton X-100 for full degradation. Both ANT4 and ATP5F1A were slightly more susceptible to pronase E in condensed than in orthodox mitochondria. Overall the data strongly support the idea that ANT4 is a protein of the inner mitochondrial membrane (Figure 5A).

Next, we studied the localization of ANT4 in nascent acrosomes of round spermatids by quantitative immune-electron microscopy (Figure 5B). The majority of gold-labeled particles were localized in the acrosome lumen. Only few particles were found near the anterior membrane and even less near the posterior membrane (Figure 5C). After further differentiation to elongated spermatids, ANT4 remained scattered across the acrosome lumen without any clear membrane association (Figure 5D). To further investigate the localization of ANT4, we released acrosome content by the acrosome reaction and separated it by sucrose density gradient centrifugation. ANT4 collected in the bottom fraction of the gradient (Figure 5E), suggesting that it was associated with dense material of the acrosome lumen, often referred to as “acrosomal matrix” (Foster et al., 2016). Likewise, acrosomal cardiolipin (TPCL) was mostly recovered in the high-density fraction of the gradient (Figure 5F). Collectively, these data suggest that ANT4 and TPCL localize to the acrosomal matrix.

Since both ANT4 and cardiolipin are associated with the inner membrane, our data specifically indicate that inner mitochondrial membranes are incorporated into the acrosome. To determine whether other mitochondrial compartments are incorporated as well, we analyzed the mitochondrial proteome present in the acrosome lumen by relative quantitative mass spectrometry. Among 334 mitochondrial proteins identified in the acrosome, 125 (37%) were annotated as matrix residents, 175 (52%) were annotated as residents of the inner membrane, 3 (1%) were annotated as residents of the intermembrane space, and 31 (9%) were annotated as residents of the outer membrane. The relative abundance of total protein in each compartment, determined by summing the mass spectrometric signal intensities of individual proteins, was in the order matrix ≈ inner membrane > outer membrane > intermembrane space, whichwas similar to the relative abundance of each compartment in mitochondria (Figure 5G). This clearly indicates that all mitochondrial sub-compartments participate in the biogenesis of acrosomes.

In summary, our data show that proteins and lipids of mitochondria, including all membranes and aqueous compartments, are assembled into the lumen of the acrosome.

## Discussion

In this paper, we confirm our previous observation that mitochondria participate in the assembly of the acrosome (Ren et al., 2019) and provide for the first time information about the mechanism. Specifically, we show that the incorporation of mitochondria into the acrosome is preceded by complex transformations that contract the matrix and alter the surface area of the inner membrane. These transformations occur during meiosis and ultimately form condensed mitochondria, in which the inner membrane-matrix is a narrow capsule that, together with the outer membrane, encloses a vacuole-like cavity. Condensed mitochondria disappear at a time when acrosomes form, suggesting that condensed mitochondria are the precursor of acrosomal mitochondria. While this conclusion is consistent with the CL patterns of these organelles, direct evidence for the specific translocation of condensed mitochondria into the acrosome is still missing.

**FIGURE 6:**
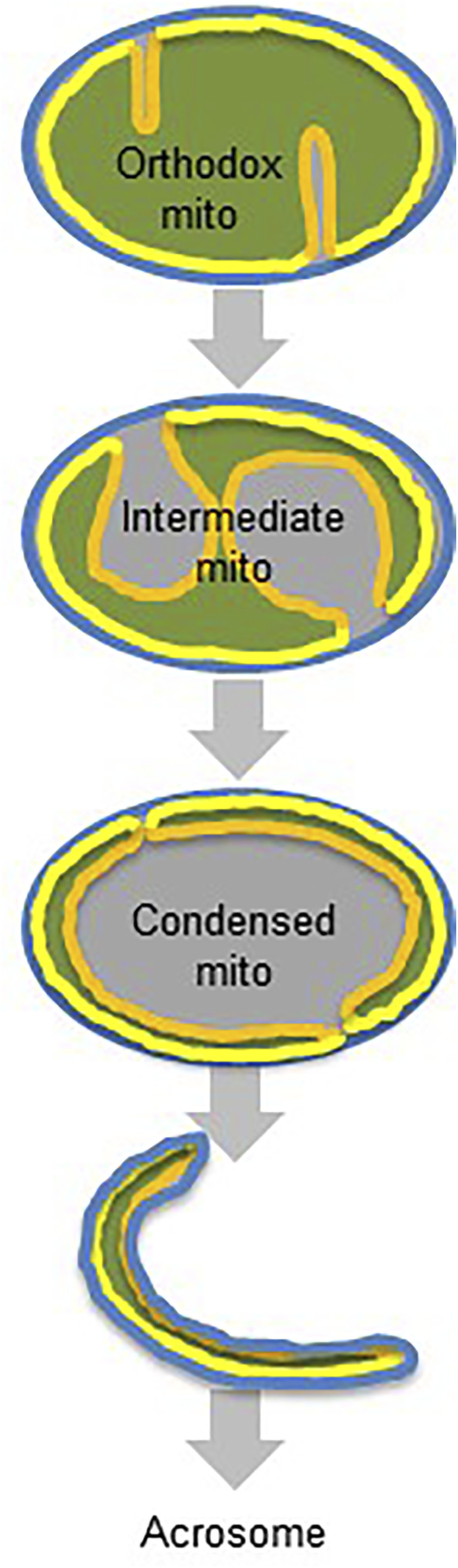
Proposed transformation of germ cell mitochondria. Orthodox mitochondria transform into intermediate mitochondria by crista membrane expansion and matrix contraction. Intermediate mitochondria transform into condensed mitochondria by contraction of the matrix and the crista membrane. Condensed mitochondria are incorporated into the acrosome lumen. Color code: blue, outer membrane; grey, intermembrane space; yellow, inner boundary membrane; orange, crista membrane; green, matrix.

In the post-meiotic period (spermiogenesis), mitochondria are incorporated into the acrosomal matrix (Figure 6). Exactly how mitochondria or their fragments end up within the acrosome lumen, remains to be established. One of the limitations of our work is that we only studied acrosomes of mature spermatozoa, which precludes inferences about the assembly mechanism. However, we show that all mitochondrial sub-compartments are translocated into the acrosome, which implies that mitochondria stay relatively intact even though they seem to transition through a stage (cytoplasmic vesicles), in which their mitochondrial morphology can no longer be recognized.

Our results have two mechanistic implications for spermiogenesis. First, they clearly demonstrate that in addition to the Golgi apparatus, mitochondria contribute to the acrosome assembly and therefore support the hypothesis that acrosomes are a lysosome-related organelle (LRO) that receives cargo from multiple intracellular sources. Several competing models have been proposed to classify the acrosome and to understand its biogenesis (Berruti, 2016; Khawar et al., 2019). The acrosome has been described alternatively as direct Golgi derivative (Friend and Fawcett, 1974), as secretory granule (Ramalho-Santos et al., 2001), as specialized lysosome, or as LRO (Berruti et al., 2010; Berruti and Paiardi, 2011). LROs are membrane-bound organelles that occur in specialized cells and are formed by the cooperation of biosynthetic (endoplasmic reticulum-Golgi) and endocytic (endosomes) pathways. For instance, melanosomes in melanocytes, lamellar bodies in type II pneumocytes, Weibel-Palade bodies in endothelial cells, and certain granules in osteoclasts or platelets, belong to the category of LRO (Raposo et al., 2007). The acrosome was proposed to be an LRO because both biosynthetic and endocytic pathways have been shown to contribute to acrosome biogenesis (Berruti et al., 2010; Berruti and Paiardi, 2011). Our data suggest that in addition to biosynthetic and endocytic pathways, mitochondria contribute to the acrosome formation.

Second, our data support the idea that mitochondria play a conserved role in the assembly of rigid structures during spermatid development. Mitochondria have been identified as precursors of the Nebenkern, a large semisolid body in insect spermatids and here we have identified mitochondria as precursors of the acrosomal matrix, another semisolid material. The function of the acrosomal matrix has not been fully understood but current ideas revolve around the need for intraluminal compartmentalization to support fertilization (Buffone et al., 2008; Khawar et al., 2019). Mitochondria may be more suited to form semisolid structures than many other organelles due to the high protein concentration in the mitochondrial matrix and the inner mitochondrial membrane (Schlame, 2021).

Beyond providing structural support, it is not clear what the functional relevance is of mitochondria in the acrosome. Testis-specific paralogs of mitochondrial proteins, such as the ANT4, the testis-specific cytochrome c, or the testis-specific ATP synthase subunit d, certainly play unique roles in the function of germ cell mitochondria either during spermatogenesis or after fertilization. For example, the testis-specific ATP synthase subunit d was shown to promote Nebenkern formation by preventing the dimerization of ATP synthase (Sawyer et al., 2017). It is possible that some of the testis-specific paralogs are essential for acrosome formation and that mitochondria play an active role in the acrosome assembly process but that remains a speculation at this point.

In conclusion, we have demonstrated that germ cell mitochondria undergo extensive transformations during spermatogenesis, which are designed in part to incorporate them into the lumen of the acrosome. The mitochondrial transformations and their involvement in acrosome biogenesis have to be seen in the context of other functions mitochondria may have during spermatogenesis, as recently described in a comprehensive review article (Wang et al., 2022).

## Data Availability

The data presented in the study are deposited in the repository MassIVE under the link https://massive.ucsd.edu/ProteoSAFe/dataset.jsp?task=0b06c8ecf5bf43fa867329a285aa776a.
